# Heterogeneous Photocatalysts Based on Nanocomposites

**DOI:** 10.3390/nano15151171

**Published:** 2025-07-30

**Authors:** Jianjun Yang, Bo Weng, Cong Wang

**Affiliations:** 1 National & Local Joint Engineering Research Center for Applied Technology of Hybrid Nanomaterials, Henan University, Kaifeng 475004, China; 2 CAS Key Laboratory of Urban Pollutant Conversion, Institute of Urban Environment Chinese Academy of Sciences, Xiamen 361021, China; bweng@iue.ac.cn; 3 School of Energy and Materials, Shihezi University, Shihezi 832003, China; 15607049970@163.com

## 1. Background

Around a decade ago, several reviews on nanocomposite photocatalysts were published [1,2,3]. By leveraging component synergy and structural optimization, nanocomposite photocatalysts have overcome the limitations of traditional single-semiconductor systems, establishing themselves as a frontier at the intersection of materials science, chemical engineering, and environmental science [4,5]. This Special Issue will advance theoretical innovation and technological translation in this field, providing support for global sustainable development.

## 2. Articles in This Special Issue

### 2.1. Research Articles

Xue et al. [6] optimized the construction of P–Bi_2_MoO_6_/g–C_3_N_4_ composites, achieving highly efficient photocatalytic bactericidal activity. The study provides a valuable reference for synthesizing environmentally friendly, solar-responsive photocatalytic sterilization materials, offering insights with regard to preparation methods, raw material selection, and structural design. Duan et al. [7] reported a MIL-derived, hollow tubular In_2_O_3_/ZnIn_2_S_4_ Z-scheme heterojunction exhibiting excellent antibacterial performance. The Z-scheme heterojunction between In_2_O_3_ and ZnIn_2_S_4_ is critical for enhancing both the degradation and antibacterial capabilities of the composite. Zhang et al. [8] developed a facile strategy for preparing *N*-doped TiO_2_ with oxygen vacancies via annealing treatment with urea, which served the dual purpose of introducing nitrogen dopants and creating oxygen vacancies in TiO_2_. Kurnosenko et al. [9] investigated photoinduced hydrogen evolution from aqueous D-glucose and D-xylose using layered perovskite-like oxides, demonstrating that organically modified derivatives of these oxides act as highly active photocatalysts for the efficient conversion of carbohydrates into hydrogen fuel.

### 2.2. Review Articles

Sun et al. [10] investigated various catalytic pyrolysis techniques for bio-oil production, focusing on the effects of different pyrolysis methods (slow, fast, and flash pyrolysis) on bio-oil yield and composition using catalysts such as zeolites, carbonaceous materials, and metal oxides. The study also highlights the correlation between catalyst properties and product distribution. Wen et al. [11] provided a comprehensive overview of the significance of transparent conducting oxide (TCO) materials in photoelectrochemical (PEC) devices, discussing their properties, fabrication methods, and current challenges. Future development directions include enhancing transparency and conductivity, advancing theoretical frameworks, and integrating multiple modification strategies to optimize TCO performance in PEC applications.

## 3. Outlook

The innovation of nanocomposite photocatalysts represents a breakthrough in materials science and a key technological milestone in advancing “carbon neutrality.” This Editorial anticipates multidisciplinary collaboration, enabling photocatalytic technologies to realize their full potential in environmental remediation, clean energy production, and medical applications, thereby contributing to global sustainable development. It also suggests that future Special Issues could focus on specific types of nanocomposite photocatalytic materials or target particular application fields to further enhance impact and relevance.
